# Amniotic Fluid-Derived Mesenchymal Stem/Stromal Cell-Derived Secretome and Exosomes Improve Inflammation in Human Intestinal Subepithelial Myofibroblasts

**DOI:** 10.3390/biomedicines10102357

**Published:** 2022-09-21

**Authors:** Hector Katifelis, Eirini Filidou, Adriana Psaraki, Farinta Yakoub, Maria G. Roubelakis, Gesthimani Tarapatzi, Stergios Vradelis, Giorgos Bamias, George Kolios, Maria Gazouli

**Affiliations:** 1 Department of Basic Medical Sciences, Laboratory of Biology, Medical School, National and Kapodistrian University of Athens, 11527 Athens, Greece; 2 Laboratory of Pharmacology, Faculty of Medicine, Democritus University of Thrace, 68100 Alexandroupolis, Greece; 3 Centre of Basic Research, Biomedical Research Foundation of the Academy of Athens, 11527 Athens, Greece; 4 Second Department of Internal Medicine, Faculty of Medicine, Democritus University of Thrace, 68100 Alexandroupolis, Greece; 5 GI Unit, Sotiria Hospital, National and Kapodistrian University of Athens, 11527 Athens, Greece; 6 2nd Department of Radiology, Medical School, National and Kapodistrian University of Athens, 12462 Athens, Greece; 7 Department of Sciences, Hellenic Open University, 26335 Patra, Greece

**Keywords:** CM, secretome, exosomes, inflammatory bowel disease, IL-1β, TLR-4, MSCs

## Abstract

Inflammatory Bowel Diseases (IBDs) are characterized by chronic relapsing inflammation of the gastrointestinal tract. The mesenchymal stem/stromal cell-derived secretome and secreted extracellular vesicles may offer novel therapeutic opportunities in patients with IBD. Thus, exosomes may be utilized as a novel cell-free approach for IBD therapy. The aim of our study was to examine the possible anti-inflammatory effects of secretome/exosomes on an IBD-relevant, in vitro model of LPS-induced inflammation in human intestinal SubEpithelial MyoFibroblasts (SEMFs). The tested CM (Conditioned Media)/exosomes derived from a specific population of second-trimester amniotic fluid mesenchymal stem/stromal cells, the spindle-shaped amniotic fluid MSCs (SS-AF-MSCs), and specifically, their secreted exosomes could be utilized as a novel cell-free approach for IBD therapy. Therefore, we studied the effect of SS-AF-MSCs CM and exosomes on LPS-induced inflammation in SEMF cells. SS-AF-MSCs CM and exosomes were collected, concentrated, and then delivered into the cell cultures. Administration of both secretome and exosomes derived from SS-AF-MSCs reduced the severity of LPS-induced inflammation. Specifically, *IL-1β*, *IL-6*, *TNF-α*, and *TLR-4* mRNA expression was decreased, while the anti-inflammatory *IL-10* was elevated. Our results were also verified at the protein level, as secretion of IL-1β was significantly reduced. Overall, our results highlight a cell-free and anti-inflammatory therapeutic agent for potential use in IBD therapy.

## 1. Introduction

Crohn’s Disease (CD) and Ulcerative Colitis (UC) belong to Inflammatory Bowel Diseases (IBDs) and are characterized by chronic relapsing inflammation of the gastrointestinal tract. Although their exact etiology remains unsolved, various factors have been implicated in playing a significant role in their pathogenesis. Such factors include environmental triggers, such as smoking, diet, and an imbalanced microbiota, immunological mediators, such as increased pro-inflammatory cytokine production, and genetic polymorphisms that enhance susceptibility to IBD [1].

Current therapies include general anti-inflammatory treatments, such as 5-Aminosalicylic Acid (5-ASA) agents, antibiotics, and corticosteroids, or more advanced and targeted approaches that include immunomodulators and biologic agents [2]. The common goal of all these treatments is to achieve long-term remission, freedom from symptoms, and improved life quality [3]. Nonetheless, not all patients respond well to treatment, with some experiencing loss of responsiveness during treatment and others lacking primary response from the beginning of therapy [4]. Therefore, there is a crucial need not only for accurate biomarkers that could predict response to treatment and indicate the most effective one, but also for new therapeutic approaches that could cover unresponsive patients.

Recently, researchers have focused on stem-cell derived therapies and, more specifically, on the use of mesenchymal stem/stromal cells (MSCs) that can be isolated from the bone marrow or adipose tissue. Such treatments may include either the administration of MSCs or their secreted factors that exhibit anti-inflammatory properties [5,6]. Specifically in CD, the administration of MSCs locally on fistula resulted in significant healing [7], while their effectiveness in refractory IBD has been under debate, as very few patients had positive clinical response following systemic infusions of MSCs [8].

However, newer evidence suggests that paracrine effects of MSCs are responsible for their anti-inflammatory role [9]. Moreover, the concept of MSC transplantation faces several challenges, including the uncertainty of the long-term effects in the recipient [10]. Thus, the study of Conditioned Medium (CM) and the contained exosomes is of utmost importance. Exosomes represent a type of lipid bilayer vesicle with a diameter of up to 150 nm. These vesicles contain bioactive molecules characteristic of the cell type from which they were derived [11]. It should also be noted that exosomes can be filter-sterilized as opposed to intact MSCs, where this is inapplicable [12]. Studies employing animal models have proposed a promising anti-inflammatory effect of exosomes in IBD and in other anti-inflammatory applications, as in the case of neuromuscular plasticity recovery [13].

In the present study, exosomes were sourced from the spindle-shaped amniotic fluid MSCs (SS-AF-MSCs), a cell population with a low immunogenic profile and enhanced proliferation rate [14]. It has been shown [15,16] that their secretome is composed of several anti-inflammatory mediators, such as the interleukins IL-10, IL-1ra, IL-13, and IL-27, along with angiogenic factors, including angiopoietin-1, PD-ECGF, uPA, and endostatin/collagen XVIII. However, it should be noted that most studies were based on animal models of IBD. An alternative in vitro system is the human intestinal subepithelial myofibroblast (SEMF). SEMFs are of mesenchymal origin, naturally reside in the lamina propria under the epithelial barrier, and except for their main role in wound healing, they also significantly contribute to other processes, such as supporting the epithelial barrier’s function and participating in immune responses [17].

Recently, we showed that SEMFs express various interleukin receptors [18], while other studies showed that SEMFs secrete proinflammatory cytokines in response to inflammatory mediators, making it an ideal model for inflammatory studies [19,20]. In a previous study [21], we showed that secretome from SS-AF-MSCs can reduce inflammation in a mouse IBD model.

Here, our aim was to investigate the potential beneficial effects of CM and exosomes using human SEMF as an in vitro IBD model. Specifically, we aimed to investigate the effects of CM/exosomes on the expression and release of various cytokines that are relevant for the pathogenesis of IBD in SEMFs treated with LPS. Moreover, we tested the effects of CM and exosomes on Toll-Like Receptor 4 (TLR-4). LPS triggers inflammation via TLR-4 [22], which has been shown to induce fibrosis in several organs, including skin and lungs, negatively affecting their function [23]. Intestinal fibrosis is a severe complication that affects both UC and CD patients [24,25], and thus, studying the actions of therapeutic molecules in its inhibition could be of great therapeutic potential. Our results provide significant evidence of their anti-inflammatory actions, allowing evaluation for future clinical applications of cell-free-based therapies in the management of IBD.

## 2. Materials and Methods

### 2.1. Patients

Colonic tissue was obtained from healthy individuals (*n* = 3) who underwent screening colonoscopy and had no pathological findings. Endoscopies were performed at the Endoscopy Department, University Hospital of Alexandroupolis, Greece. The local Research Ethics Committee approved this study, and patients gave their informed written consent before participation.

### 2.2. Isolation, Characterization, and Culture of Human Intestinal Subepithelial Myofibroblasts

**Figure 1:**
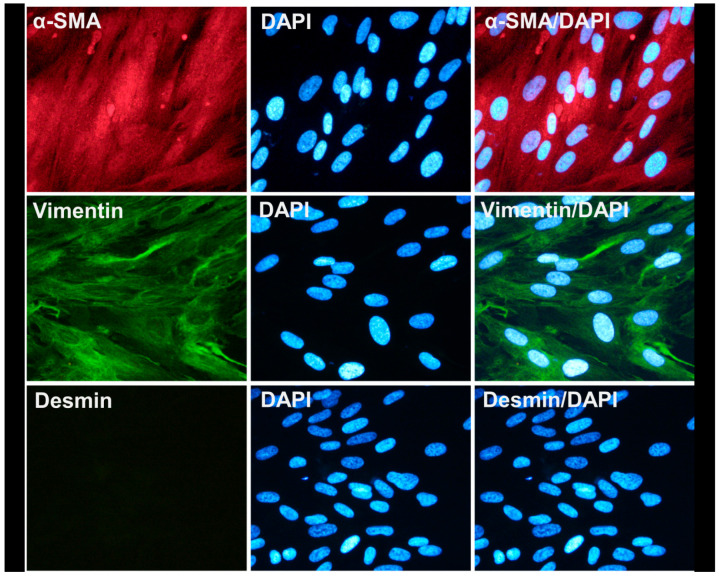
Characterization of isolated colonic subepithelial myofibroblasts. Representative images of colonic subepithelial myofibroblasts that were found positive for α-SMA (red) and vimentin (green) expression and negative for desmin expression. Nuclei were stained with DAPI (blue). Magnification was set at 400×.

SEMFs were isolated from colonic biopsies, as previously described [14]. Briefly, the collected colonic biopsies were washed and de-epithelialized using dithiothreitol 1 mM (DTT; Sigma-Aldrich, Darmstadt, Germany) and ethylene-diaminetetraacetic acid 1 mM (EDTA; Sigma-Aldrich, Darmstadt, Germany). Tissue denuded of epithelial cells was then transferred and cultured in Dulbecco’s Modified Eagle Medium (DMEM; Biosera, Nuaille, France) plus 10% fetal bovine serum (FBS; Biosera, Nuaille, France) in 5% CO_2_ at 37 °C. During culture, numerous nonadherent and adherent cells appeared in the culture flasks, and their culture medium was changed every 72 h. Denuded mucosal tissue was maintained in culture for up to 4 weeks, until numerous foci of myofibroblasts adhered on the flask surface. Tissue specimens were then removed, and intestinal myofibroblasts were characterized using immunofluorescence microscopy as being α-smooth muscle actin (α-SMA)- and vimentin-positive and desmin-negative, as shown in Figure 1.

Colonic subepithelial myofibroblasts at passages 2–5 were used in these studies. All experiments were performed with FBS-free media at 95% culture confluence with a stable ratio of supernatant volume-to-surface available for cell adhesion (1.5 mL:9.6 cm^2^). Colonic subepithelial myofibroblasts were passaged at a ratio of 1:3.

### 2.3. CM Preparation

CM preparation was performed as previously described [16,21]. One-and-a-half million AF-MSCs were seeded until 80–90% confluence and were further cultured with DMEM (Thermo Fisher Scientific Inc. Gibco, 41966-029, New York, NY, USA) supplemented with 0.5% FBS (Thermo Fisher Scientific Inc, Gibco, 10500064, New York, NY, USA) for 48 h. CM was then collected and centrifuged at 1200× *g* for 5 min to remove cell debris, and supernatant was concentrated at approximately 25-fold using ultrafiltration columns with a 3 KDa cut-off filter (Amicon Ultra 3K Millipore, UFC900308, Bedford, MA, USA).

### 2.4. Exosome Isolation, Western Blot Characterization, and Transmission Electron Microscopy (TEM) for Exosome Imaging

Exosomes were collected from 7.5 × 10^6^ AF-MSCs cultured in DMEM for 48 h until 90% confluency (Gibco-BRL, Paisley, UK) with 0.5% exosome-depleted FBS (Gibco-BRL, Paisley, UK), as described in our previous publications [16,26,27,28]. All samples were collected with a written informed consent, following the Ethical Committee approval of Alexandra Hospital, Athens, Greece, the bioethics committee of the School of Medicine of the NKUA, and the BRFAA. Spindle-shaped colonies from 2 samples of AF-MSCs were selected for subculture in the present study, and cells at 10–20 passages until 80% confluency were used for exosome collection [26,29].

The media had been previously centrifuged at 37,500 rpm for 16 h to eliminate the FBS-derived exosomes. The (CM) was collected and centrifuged at 1000× *g* for 5 min to remove cell debris and then at 3225× *g* for 15 min to remove apoptotic bodies. The supernatant was then concentrated at 20 mL, using ultrafiltration columns with 3 KDa cutoff units, and centrifuged (SORVALL 100SE Floor Ultra Speed Centrifuge in Watertown, MA, USA-number of rotor T865) at 37,500 rpm for 2 h at 4 °C. Pellets were resuspended in 50 μL PBS (Thermo Fisher Scientific Inc. Gibco, USA) and stored at −80 °C. The supernatant was also concentrated with 3 KDa columns, filtered with 0.2 μm filters (AHLSTROM, Helsinki, Filand), and stored at 4 °C to be used as a negative control. Protein concentration was calculated by Bradford assay (BioRad Laboratories Inc., Hercules, CA, USA) with an average concentration of 0.2 μg/μL.

Western blotting was performed to evaluate the exosome isolation from AF-MSC-CM. Exosomal proteins with an average of 15 μg were separated using 12% SDS-PAGE gels, transferred to PVDF (Immun-Blot PVDF membrane for Protein Blotting, BIO-RAD, CA, USA), and blocked in 5% milk in TBST (1× Tris-Buffered Saline, 0.1% Tween) for 1 h at room temperature. The membranes were incubated with mouse anti-Flotilin1 (1:1000, sc-133153, Santa Cruz, CA, USA), mouse anti-CD63 (1:500, SC-5275, Santa Cruz, CA, USA), mouse anti-CD9 (1:200, sc-13118, Santa Cruz, CA, USA), and mouse anti-GAPDH (1:1000, MAB374, Millipore, Burlington, MA, USA) antibodies overnight at 4 °C. Three washes with TBST (1× Tris-Buffered Saline and 1% Tween) followed, and membranes were incubated with anti-mouse HRP-conjugated secondary antibody (1:1000, Millipore, USA) for 1 h. Blots were washed 2 times with TBST and 1 time with TBS alone for 10 min, and the expression signals were visualized by a Western blot detection system (iBright CL1500 Imaging System, MA, USA) using ECL (Luminata Forte, Millipore, MA, USA). Exosome-depleted, concentrated, and filtered CM was used as negative control (AF-MSC-EXO-CONTROL).

The exosome samples derived from AF-MSC cells were fixed 1:1 with 4% paraformaldehyde overnight at 4 °C. A volume of 5 µL of fixed samples was placed onto 300 mesh copper grids with carbon-coated formvar film and incubated for 20 min. Brief PBS washes (Thermo Fisher Scientific Inc. Gibco, NY, USA) followed, and grids were incubated with 1% glutaraldehyde for 5 min and washed with dH_2_O. Grids were stained with uranyl oxalate (pH 7) for 5 min and methyl cellulose-uranyl acetate for 10 min on ice. The excess liquid was removed by Whatman filter paper, and the grids were allowed to dry. Samples were imaged with a Philips 420 Transmission Electron Microscope at an acceleration voltage of 60 kV and photographed with a Megaview G2 CCD camera (Olympus SIS, Münster, Germany).

### 2.5. Inflammation Induction and CM/Exosome Incubation and RNA Extraction

Cells were transferred to 6-well plates with a concentration of 25,000 cells/mL. Four different conditions were used: (A) Cells that were only incubated with LPS (100 ng) overnight; (B) Cells that were incubated with LPS (100 ng) and CM (500 μL) overnight; (C) Cells that were incubated with LPS (100 ng) and exosomes (48 μL) overnight; (D) Cells that were not incubated to LPS, CM, or exosomes that were used as a control (untreated cells). Subsequently, nutrient media were removed, and cells were rinsed once with PBS. RNA was extracted using the NucleoZOL (Macherey-Nagel, Düren, German) method, according to the manufacturer’s instructions.

### 2.6. cDNA Synthesis and Real Time PCR

cDNA synthesis was performed using a PrimeScript First-Strand cDNA kit (Takara Bio Europe SAS, Saint-Germain-en-Laye, France). One microgram of RNA was incubated for 30 min at 37 °C, and 5 s incubation at 85 °C followed, in a reaction that contained 500 μg of Oligo dT, 10 mM deoxyribo-nucleotide triphosphates 5× first-strand buffer 0.1 M dithiothreitol, and 200 U/mL reverse transcriptase. Then, mRNA fold change was estimated using GAPDH as a gene of reference. These mRNAs correspond to *IL-1β*, *IL-6*, *IL-8*, *IL-10*, *ANXA1*, *TNF-α*, and *TLR-4.* Quantitative real-time PCR was performed on an ABI Prism apparatus (Applied Biosystems, Foster City, CA, USA). Gene expression levels were normalized by subtracting Ct value of the GAPDH RNA from that of GOI using the equation (ΔCt = −|CtGOI − CtGAPDH|). Relative expression of *IL-1β*, *IL-6*, *IL-8*, *IL-10*, *ANXA1*, *TNF-α*, and *TLR-4* was determined comparing the samples from cells that were incubated with LPS/LPS + CM/LPS + Exosomes with untreated cells, using the 2^−ΔΔCt^ model in which ΔΔCt = ΔCt_GOI_ − ΔCt_GAPDH_. Two biological replicates with three technical replicates each were used to ensure reproductivity. The primers used for these reactions are shown in Table 1.

### 2.7. Extracellular IL-1β Quantification

Extracellular IL-1β levels were quantified (after incubation with 100 ng of LPS, incubation of 100 ng of LPS and CM, incubation of 100 ng of LPS and Exosomes, and after no LPS/CM/Exosome incubation) in SEMF cells using the ELISA technique (Cusabio, Houston, TX, USA). Absorbance was read at 450 nm, and the average zero standard optical density (OD) was subtracted from the samples’ and standard ODs. All samples were held in duplicate and under the manufacturer’s instructions.

### 2.8. Statistical Analysis

Statistically significant differences between the values of the samples were evaluated by one-way analysis of variance (ANOVA) using GraphPad version 3.00 (GraphPad Software, San Diego, CA, USA). *p* < 0.05 value was considered statistically significant.

## 3. Results

### 3.1. Exosome Characterization

**Figure 2:**
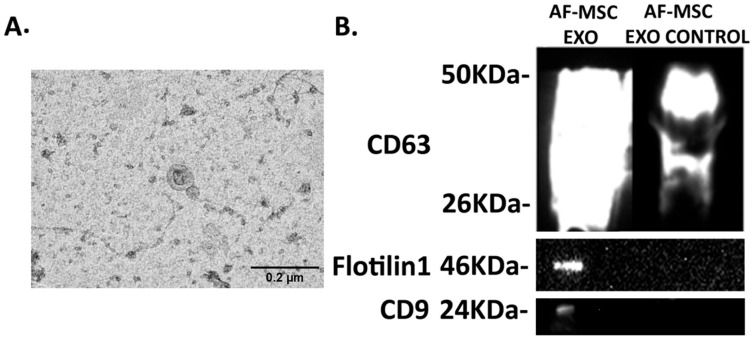
AF-MS-EXO characterization: (**A**) The size of exosomes was confirmed using TEM. Scale bar 0.2 μm. Original magnification 60,000×. (**B**) The expression of CD63, Flotilin1, and CD9 was detected by Western blotting. AF-MSC-EXO: AF-MSC-exosomes; AF-MSC-EXO control: AF-MSC-CM that is deprived of exosomes, concentrated, and filtered.

The successful isolation of AF-MSC-Exosomes was confirmed using TEM and Western blotting. As seen in Figure 2, the average size of AF-MSC-EXOs was measured to be approximately 100 nm (Figure 2A), and the positive protein expression of the exosomal markers—CD63, Flotilin 1, and CD9—was detected in AF-MSC-EXO samples compared to AF-MSC-EXO control (Figure 2B).

### 3.2. Characterization of Human Intestinal Subepithelial Myofibroblasts

Having successfully isolated and characterized the AF-MSC-Exosomes, we proceeded in the isolation and characterization of human intestinal subepithelial myofibroblasts (SEMFs, in order to develop an in vitro inflammation model for studying the possible therapeutic effect of the exosomes. As already described in the Materials and Methods section, we successfully isolated human intestinal SEMFs from colonic biopsies, and these cells were found to express vimentin and α-SMA, but not desmin (Figure 1). This staining pattern is in agreement with the current literature [17,30], indicating that these primarily isolated cells were subepithelial myofibroblasts.

### 3.3. Confirmation of Inflammation Model

**Figure 3:**
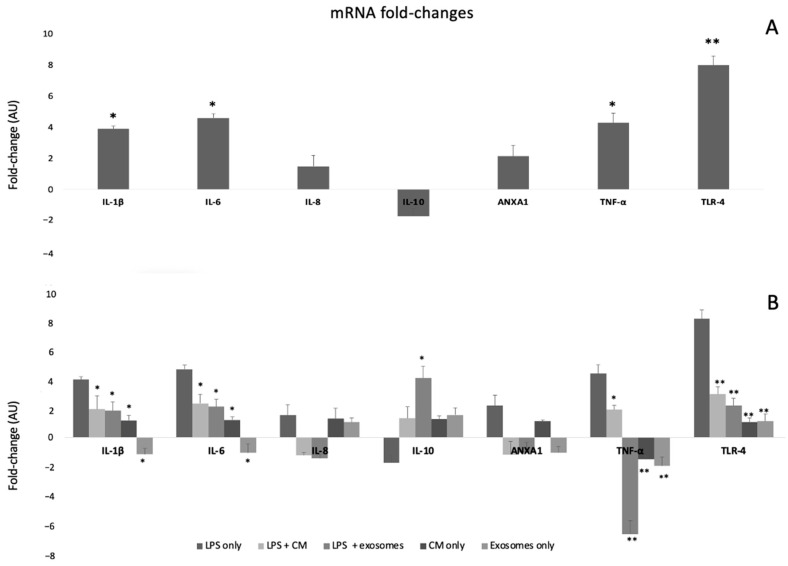
(**A**) Fold changes of genes in SEMF cells that were incubated with LPS compared to cells that received no LPS treatment. The asterisk symbols (*, **) indicate statistical significance (*p* < 0.05 and *p* < 0.01, respectively) using one-way ANOVA compared to cells that were not treated with LPS. (**B**) Fold changes of genes in SEMF cells that were incubated with LPS, LPS + CM, LPS + exosomes, CM (without LPS), or exosomes (without LPS). The asterisk symbols (*, **) indicate statistical significance (*p* < 0.05 and *p* < 0.01, respectively) using one-way ANOVA between cells treated with LPS + CM, LPS + Exosomes, CM, or exosomes and LPS-only treated cells.

As indicated in Figure 3A, incubation of SEMFs with LPS (100 ng, overnight) led to a statistically significant increase in the following pro-inflammatory genes: *IL-1β*, (3.90-fold change, *p* < 0.05), *IL-6* (4.60-fold change, *p* < 0.05), *TNF-α* (4.30-fold change, *p* < 0.05), and *TLR-4* (8.00-fold change, *p* < 0.01) compared to cells that were not incubated with LPS, confirming the successful development of the in vitro SEMF inflammation model. Both *IL-8* and *ANXA1* showed a non-statistically significant upregulation. On the contrary, the anti-inflammatory *IL-10* showed a downregulation (not statistically significant).

### 3.4. Therapeutic Effects of CM and Exosomes

Incubation of SEMFs with LPS and CM led to statistically significant lower values of the inflammatory markers *IL-1β* (1.90-fold change, *p* < 0.05), *IL-6* (2.30-fold change), *TNF-α* (1.87-fold change), and *TLR-4* (2.92-fold change, *p* < 0.01) than those of the LPS-only treated cells, suggesting that CM, indeed, has an anti-inflammatory effect. Both *IL-8* and *ANXA1* showed a mild downregulation that was not statistically significant. Regarding *IL-10*, a non-statistically significant upregulation was observed.

Quite similarly, incubation with LPS and exosomes also showed lower values of *IL-1β* (1.80-fold change, *p* < 0.05), *IL-6* (2.07-fold change), *TNF-α* (−7.00-fold change, *p* < 0.05), and *TLR-4* (2.14-fold change, *p* < 0.01). Regarding *IL-8* and *ANXA1*, a mild but not statistically significant downregulation was observed. However, unlike CM treatment, exosomes also led to the upregulation of the anti-inflammatory *IL-10* (4.01-fold change, *p* < 0.05), as shown in Figure 3B, a finding that suggests more potent anti-inflammatory properties.

Finally, treatment with CM or exosomes (without LPS incubation) had no statistically significant change in any of the studied genes, as shown in Figure 3B.

### 3.5. Extracellular IL-1β

**Figure 4:**
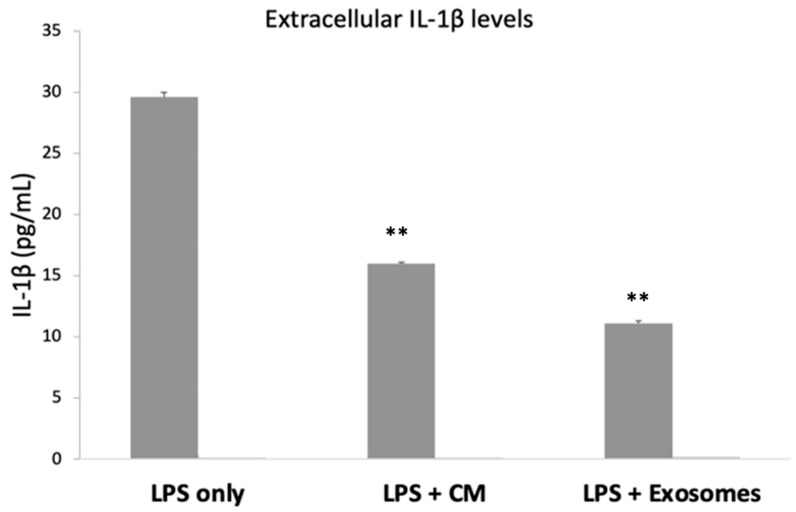
Extracellular levels of IL-1β in SEMF cells treated with LPS, LPS with CM, and LPS with Exosomes. IL-1β from untreated cells (no LPS, CM, or Exosomes) was undetectable. The asterisk symbols (**) indicate statistical significance (*p* < 0.01, respectively) using one-way ANOVA compared to cells treated with LPS.

Having confirmed that both the CM and the exosomes have a statistically significant anti-inflammatory effect on the mRNA levels of various studied cytokines, we proceeded with measuring the protein levels of IL-1β, as it is a very potent pro-inflammatory mediator. Overnight incubation with LPS resulted in the secretion/release of IL-1β (29.61 ± 0.4 pg/mL). When SEMFs were exposed to both LPS and CM, the protein levels of IL-1β were reduced (15.96 ± 0.1 pg/mL, *p* < 0.01), and a similar reduction was also observed in SEMFs that were exposed to LPS and exosomes (11.11 ± 0.2 pg/mL, *p* < 0.01), suggesting that both CM and exosomes, indeed, have a strong anti-inflammatory effect. The extracellular IL-1β in the untreated cells was undetectable. The results are shown in Figure 4.

## 4. Discussion

In the current study, we used human intestinal SEMFs as a disease model for the investigation of the anti-inflammatory effects of CM and exosomes. Our work offers novel findings for the role of exosomes, as we demonstrate that both the CM and the exosomes statistically significantly downregulated the mRNA expression of the LPS-induced inflammation-related genes (*TNF-α*, *IL1-β*, *IL-6)* in SEMFs, and this downregulation was also confirmed at the protein level of IL-1β, highlighting a possible anti-inflammatory therapeutic strategy for IBD.

Even though the etiology of IBD remains obscure, it is well-understood that the epithelium is capable of secreting and responding to a plethora of immunological mediators, and that it is a major participant in IBD pathophysiology [31]. Quite recently, Yang et al. [32] showed that exosomes could be useful in tissue regeneration since they repair lost intestinal barrier integrity. However, although exosomes have been studied in the context of IBD [33,34], the literature is scarce regarding the effect of exosomes on inflammatory markers of human SEMFs.

Herein, we showed that *TNF-α*, *IL-1β*, and *IL-6* expression was reduced at the mRNA level in SEMFs incubated with CM and exosomes treatment compared to the LPS-only treated cells (Figure 3B). These findings are in accordance with our previous study [21], in which we showed that SS-AF-MSC secretome reduces inflammation in a mouse model of IBD. Comparing the effects of CM and exosomes, the most notable differences were in the *TNF-α*, *IL-1β*, and *IL-6* gene expression when cells were treated with exosomes. The role of these cytokines is well-established in IBD, particularly of *TNF-α*, against which several biological agents have been developed over the past 25 years [35] with satisfactory results. However, the use of anti-TNF agents is oftentimes compromised by infusion perfusion reactions and the risk of severe infections [36]. Thus, there is a constant need for additional anti-inflammatory treatments to be available. Moreover, the notion of suppressing TNF-α has further expanded to IL-6 and IL-8 as pharmaceutical means of reducing inflammation. Both anti-IL-6 [37] treatment and IL-8 antagonists [38] have been used as alternatives to the use of corticosteroids and TNF-α inhibitors in the hope of fewer side effects. Based on our results, both CM and exosomes can reduce these inflammatory cytokines. Additionally, the current literature demonstrates the low immunogenicity of SS-AF-MSC secretome [39], while at the same time the CM/exosomes offer a cell-free alternative treatment based on paracrine effect and not on engraftment or differentiation [10].

Regarding the mRNA levels of the anti-inflammatory *IL-10*, an increase after CM/exosome treatment was observed (Figure 3B). This is an important aspect of the CM/exosome action, since *IL-10* decrease is linked to IBD; in particular, Il-10 (−/−) mice develop enterocolitis [40]. More interestingly, efforts have been made for IL-10 supplementation but with poor results [40]. However, both CM and exosomes managed to increase the mRNA levels of this cytokine, and this effect further substantiates their anti-inflammatory profile.

To elucidate the effects of CM/exosomes, IL-1β levels were further quantified at protein levels (Figure 4). In accordance with the upregulation of *IL-1β*, secreted IL-1β levels showed a concomitant increase after LPS incubation, while the combination of either LPS and CM or LPS and exosomes resulted in decreased levels. This is important since IL-1β acts as a potent inflammatory molecule that is already associated with IBD severity. In addition, pharmaceutical agents that target IL-1β have been already tested (including anti-IL-1β antibodies) [41]. Furthermore, this finding is in accordance with our previous study [21], where the protein levels of TNF-α and TGF-β were found to be reduced upon CM exposure in a mouse model of colitis.

Based on our results, exosomes (and to a lesser extent CM) emerged as possible candidate agents that could reduce IL-1β secretion/release and consequently ameliorate IBD management.

Finally, *TLRP-4* expression was also reduced in cells exposed to LPS after CM or exosome incubation. This is an important finding since it expands the potential of the tested CM/exosome to the possible inhibition of fibrosis. *TLR-4* has been found to be upregulated upon LPS exposure [22], while it has been found to promote fibrosis in several organs [23]. In the context of CD and UC, fibrosis represents severe complication, while it is also serves as an indication of patients unresponsive to treatment [24,25]. Thus, pharmaceutical agents that could inhibit this progress could be of great clinical interest.

Regarding the differences between the anti-inflammatory effects of CM and exosomes, the latter have a more potent anti-inflammatory effect, as shown in the reduction in secreted IL-1β (Figure 4) and TNF-α and the increase in IL-10 (Figure 3B). Thus, exosomes may represent potential therapeutic agents towards IBD, a finding that is consistent with the anti-inflammatory effects observed in other studies [39,40]. Moreover, the cell-free nature of exosome treatment, which does not bear the theoretical dangers of MSC grafts [10], combined with the advantage that exosomes can also be sterilized further, highlights the therapeutic potential of exosomes [12]. However, several challenges remain, including estimating the therapeutic dosage and the current means of producing them in adequate quantities [42].

## 5. Conclusions

In conclusion, exosomes and, to a lesser extent, CM treatment in SEMFs show a significant reduction in pro-inflammatory responses, as shown by the reduction in the expression of the pro-inflammatory markers *IL-1β*, *IL-6*, and *TNF-a* and the increased expression of the anti-inflammatory *IL-10* (compared to LPS-treated SEMFs). The extracellular levels of IL-1β are also reduced. In parallel, reduction in *TLR-4* further highlights the anti-inflammatory potential of CM and exosomes as agents that could be used as a cell-free therapy for the management of IBD. However, further experimental approaches are warranted to specifically identify their mechanisms of action. Future studies are required to confirm and further expand these findings.

## Figures and Tables

**Table 1:** Sequences of primers used.

Gene	Forward Primer	Reverse Primer
**IL-1β**	5′-CCTTGTCGAGAATGGGCAGT-3′	5′-TCCTGTCGACAATGCTGCCT-3′
**IL-6**	5′-CCAGCTATGAACTCCTTCT-3′	5′-GCTTGTTCCTCACATCTCT-3′
**IL-8**	5′-ACTGAGAGTGATTGAGAGTGGAC-3′	5′-AACCCTCTGCACCCAGTTTTC-3′
**IL-10**	5′-TCTCCGAGATGCCTTCAGCAGA-3′	5′-TCAGACAAGGCTTGGCAACCCA-3′
**ANXA1**	5′-GCAGGCCTGGTTTATTGAAA-3′	5′-GCTGTGCATTGTTTCGCTTA-3′
**TNF-α**	5′-CACGTCGTAGCAAACCACCAAGTGG-3′	5′-GATAGCAAATCGGCTGACGGTGTGG-3′
**TLR-4**	5′-GATTAGCATACTTAGACTAC-3′	5′-GATCAACTTCTGAAAAAGCATTCCCAC-3′
**GAPDH**	5′-TTCACCACCATGGAGAAGGC-3′	5′-GGCATGGACTGTGGTCATGA-3′

## Data Availability

The original contributions presented in the study are included in the article. Further inquiries can be directed to the corresponding author.
